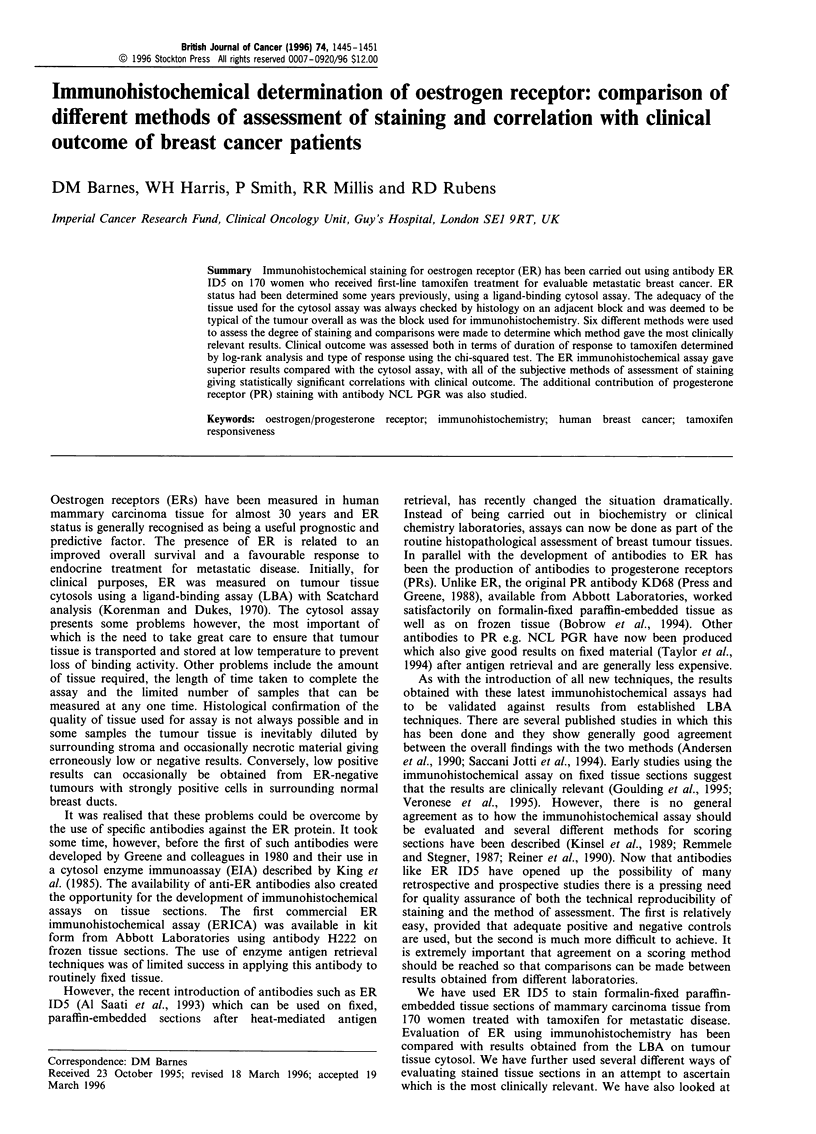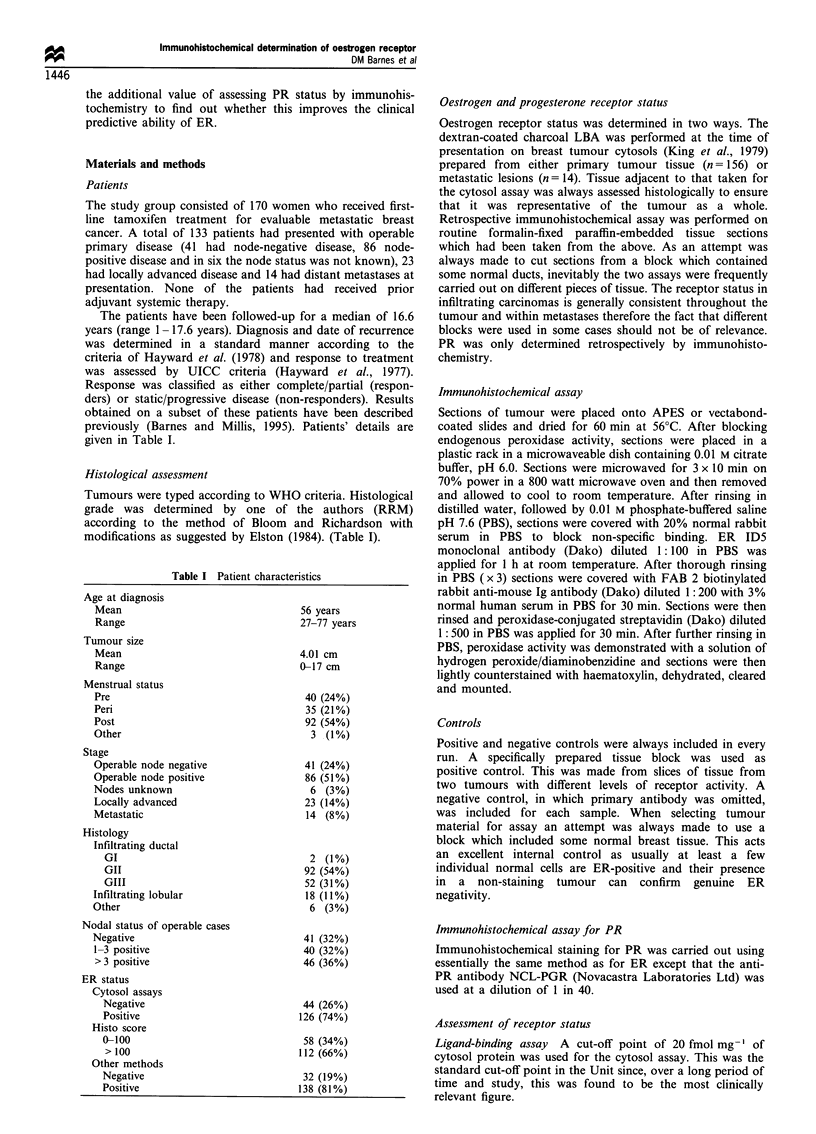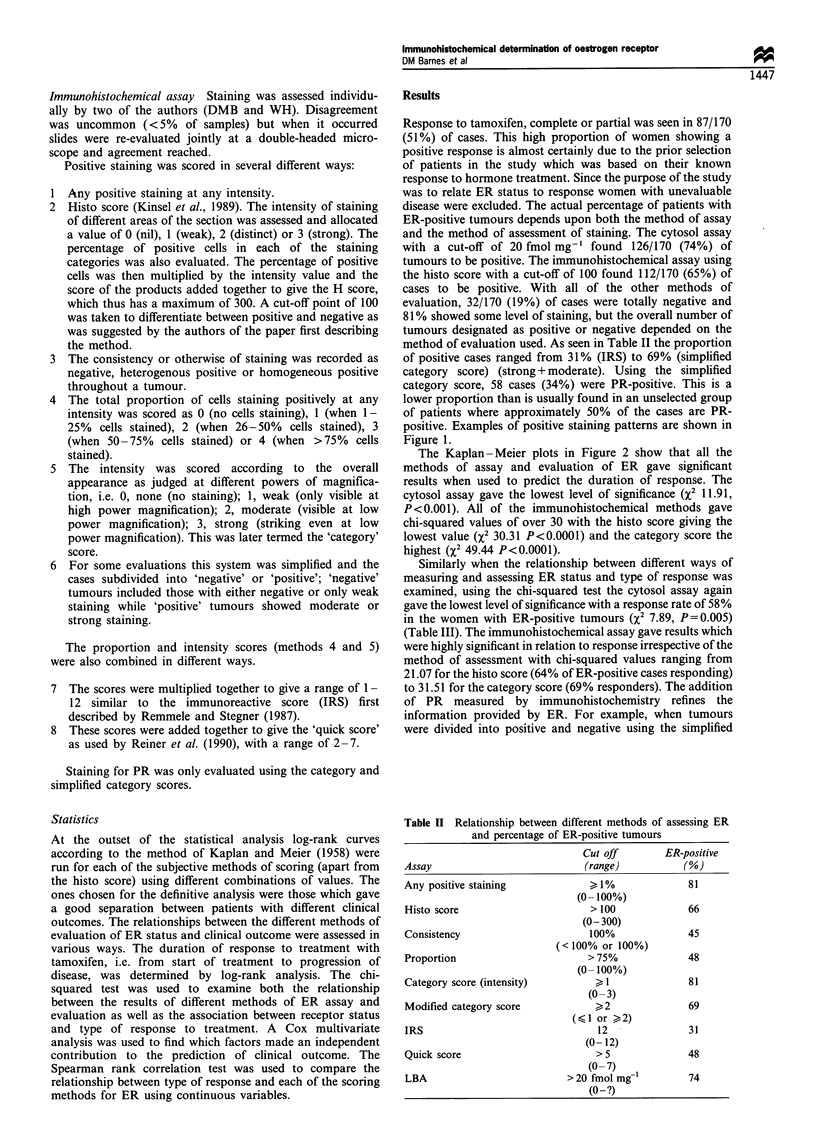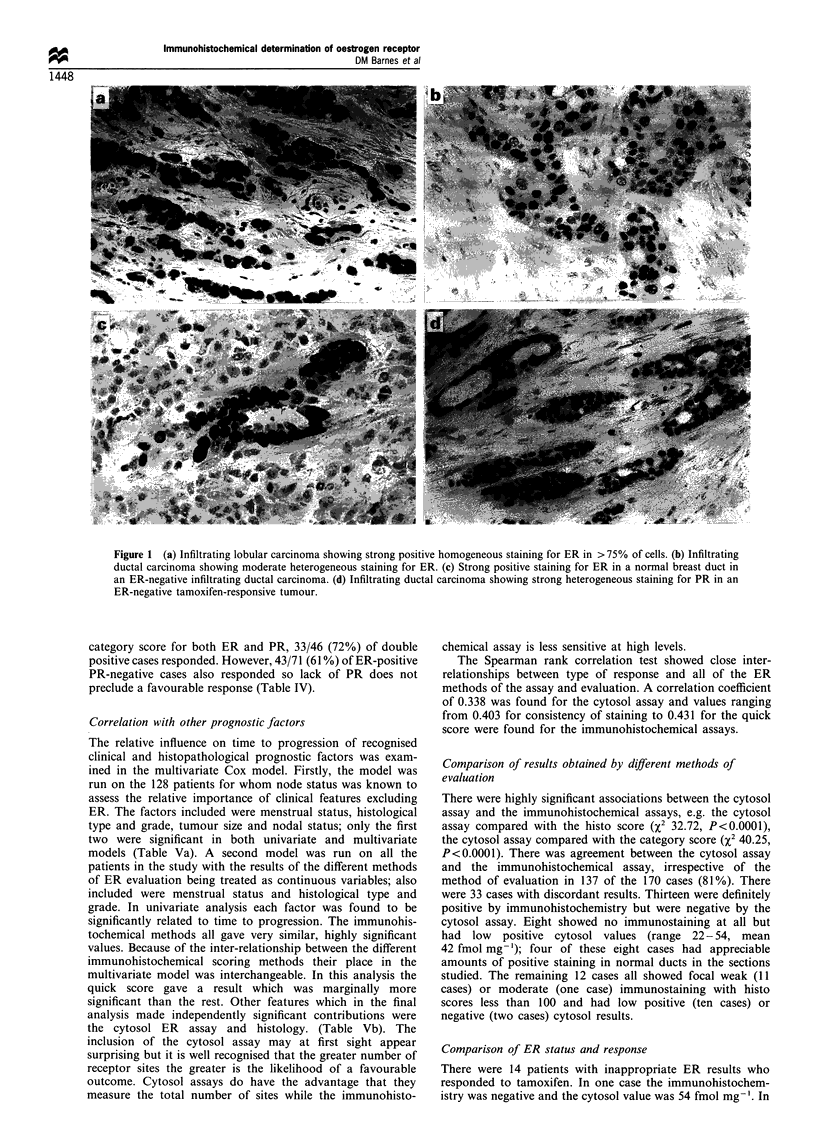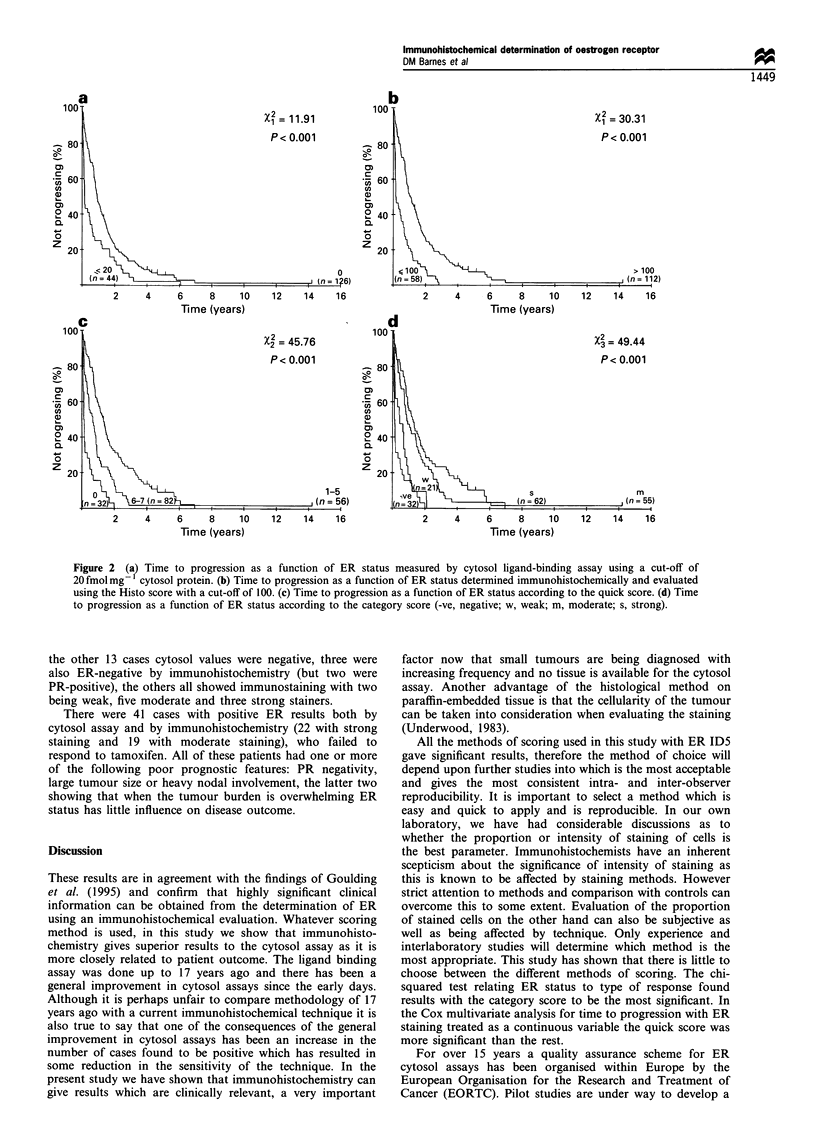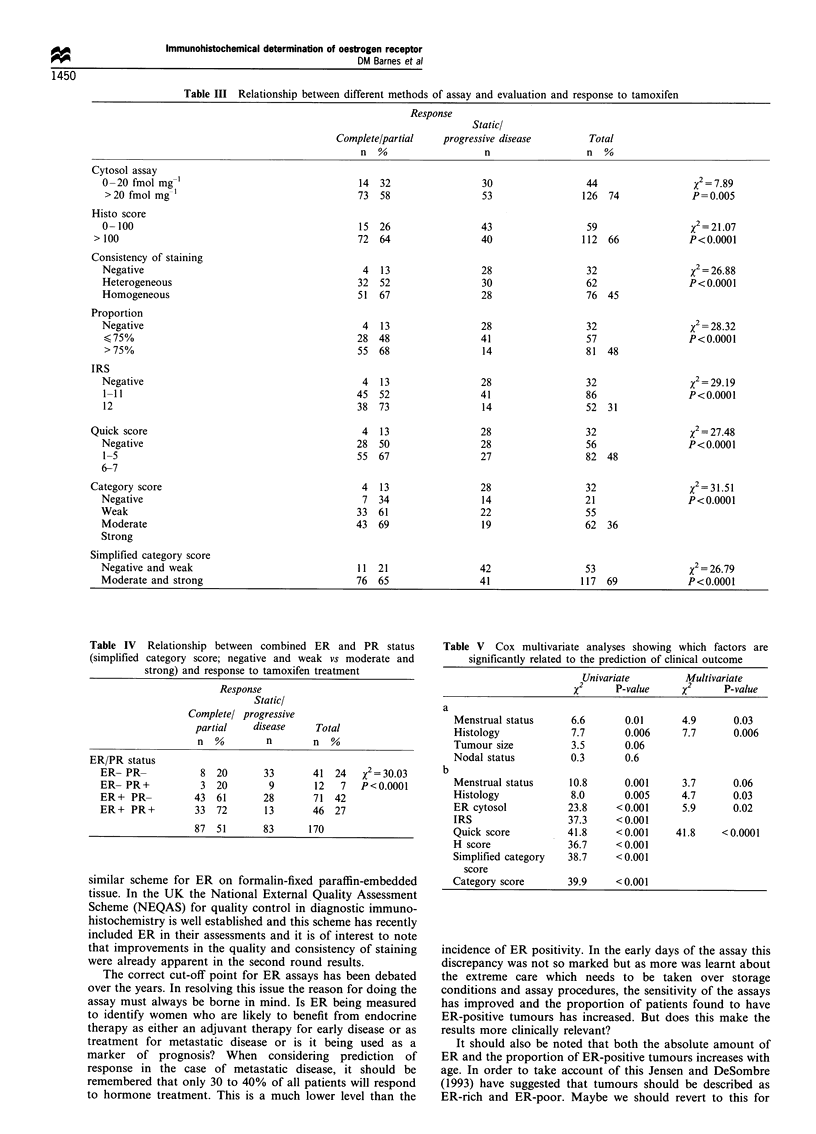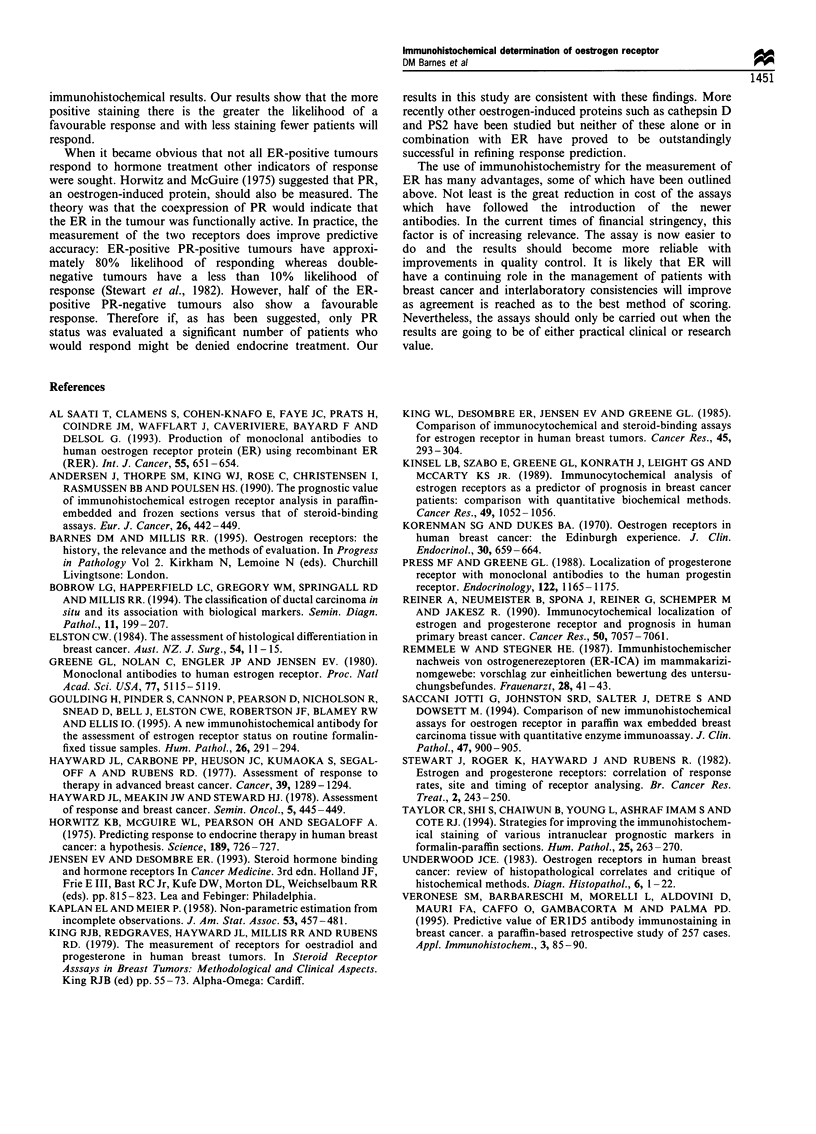# Immunohistochemical determination of oestrogen receptor: comparison of different methods of assessment of staining and correlation with clinical outcome of breast cancer patients.

**DOI:** 10.1038/bjc.1996.563

**Published:** 1996-11

**Authors:** D. M. Barnes, W. H. Harris, P. Smith, R. R. Millis, R. D. Rubens

**Affiliations:** Imperial Cancer Research Fund, Guy's Hospital, London, UK.

## Abstract

**Images:**


British Journal of Cancer (1996) 74, 1445-1451
© 1996 Stockton Press All rights reserved 0007-0920/96 $12.00

### Immunohistochemical determination of oestrogen receptor: comparison of different methods of assessment of staining and correlation with clinical outcome of breast cancer patients

DM Barnes, WH Harris, P Smith, RR Millis and RD Rubens

*Imperial Cancer Research Fund, Clinical Oncology Unit, Guy's Hospital, London SE1 9RT, UK*

**Summary** Immunohistochemical staining for oestrogen receptor (ER) has been carried out using antibody ER ID5 on 170 women who received first-line tamoxifen treatment for evaluable metastatic breast cancer. ER status had been determined some years previously, using a ligand-binding cytosol assay. The adequacy of the tissue used for the cytosol assay was always checked by histology on an adjacent block and was deemed to be typical of the tumour overall as was the block used for immunohistochemistry. Six different methods were used to assess the degree of staining and comparisons were made to determine which method gave the most clinically relevant results. Clinical outcome was assessed both in terms of duration of response to tamoxifen determined by log-rank analysis and type of response using the chi-squared test. The ER immunohistochemical assay gave superior results compared with the cytosol assay, with all of the subjective methods of assessment of staining giving statistically significant correlations with clinical outcome. The additional contribution of progesterone receptor (PR) staining with antibody NCL PGR was also studied.

**Keywords:** oestrogen/progesterone receptor; immunohistochemistry; human breast cancer; tamoxifen responsiveness

Oestrogen receptors (ERs) have been measured in human mammary carcinoma tissue for almost 30 years and ER status is generally recognised as being a useful prognostic and predictive factor. The presence of ER is related to an improved overall survival and a favourable response to endocrine treatment for metastatic disease. Initially, for clinical purposes, ER was measured on tumour tissue cytosols using a ligand-binding assay (LBA) with Scatchard analysis (Korenman and Dukes, 1970). The cytosol assay presents some problems however, the most important of which is the need to take great care to ensure that tumour tissue is transported and stored at low temperature to prevent loss of binding activity. Other problems include the amount of tissue required, the length of time taken to complete the assay and the limited number of samples that can be measured at any one time. Histological confirmation of the quality of tissue used for assay is not always possible and in some samples the tumour tissue is inevitably diluted by surrounding stroma and occasionally necrotic material giving erroneously low or negative results. Conversely, low positive results can occasionally be obtained from ER-negative tumours with strongly positive cells in surrounding normal breast ducts.

It was realised that these problems could be overcome by the use of specific antibodies against the ER protein. It took some time, however, before the first of such antibodies were developed by Greene and colleagues in 1980 and their use in a cytosol enzyme immunoassay (EIA) described by King *et al.* (1985). The availability of anti-ER antibodies also created the opportunity for the development of immunohistochemical assays on tissue sections. The first commercial ER immunohistochemical assay (ERICA) was available in kit form from Abbott Laboratories using antibody H222 on frozen tissue sections. The use of enzyme antigen retrieval techniques was of limited success in applying this antibody to routinely fixed tissue.

However, the recent introduction of antibodies such as ER ID5 (Al Saati *et al.*, 1993) which can be used on fixed, paraffin-embedded sections after heat-mediated antigen retrieval, has recently changed the situation dramatically. Instead of being carried out in biochemistry or clinical chemistry laboratories, assays can now be done as part of the routine histopathological assessment of breast tumour tissues. In parallel with the development of antibodies to ER has been the production of antibodies to progesterone receptors (PRs). Unlike ER, the original PR antibody KD68 (Press and Greene, 1988), available from Abbott Laboratories, worked satisfactorily on formalin-fixed paraffin-embedded tissue as well as on frozen tissue (Bobrow *et al.*, 1994). Other antibodies to PR e.g. NCL PGR have now been produced which also give good results on fixed material (Taylor *et al.*, 1994) after antigen retrieval and are generally less expensive.

As with the introduction of all new techniques, the results obtained with these latest immunohistochemical assays had to be validated against results from established LBA techniques. There are several published studies in which this has been done and they show generally good agreement between the overall findings with the two methods (Andersen *et al.*, 1990; Saccani Jotti *et al.*, 1994). Early studies using the immunohistochemical assay on fixed tissue sections suggest that the results are clinically relevant (Goulding *et al.*, 1995; Veronese *et al.*, 1995). However, there is no general agreement as to how the immunohistochemical assay should be evaluated and several different methods for scoring sections have been described (Kinsel *et al.*, 1989; Remmele and Stegner, 1987; Reiner *et al.*, 1990). Now that antibodies like ER ID5 have opened up the possibility of many retrospective and prospective studies there is a pressing need for quality assurance of both the technical reproducibility of staining and the method of assessment. The first is relatively easy, provided that adequate positive and negative controls are used, but the second is much more difficult to achieve. It is extremely important that agreement on a scoring method should be reached so that comparisons can be made between results obtained from different laboratories.

We have used ER ID5 to stain formalin-fixed paraffin-embedded tissue sections of mammary carcinoma tissue from 170 women treated with tamoxifen for metastatic disease. Evaluation of ER using immunohistochemistry has been compared with results obtained from the LBA on tumour tissue cytosol. We have further used several different ways of evaluating stained tissue sections in an attempt to ascertain which is the most clinically relevant. We have also looked at

Correspondence: DM Barnes
Received 23 October 1995; revised 18 March 1996; accepted 19 March 1996

the additional value of assessing PR status by immunohistochemistry to find out whether this improves the clinical predictive ability of ER.

### Materials and methods

### *Patients*

The study group consisted of 170 women who received first-line tamoxifen treatment for evaluable metastatic breast cancer. A total of 133 patients had presented with operable primary disease (41 had node-negative disease, 86 node-positive disease and in six the node status was not known), 23 had locally advanced disease and 14 had distant metastases at presentation. None of the patients had received prior adjuvant systemic therapy.

The patients have been followed-up for a median of 16.6 years (range 1 – 17.6 years). Diagnosis and date of recurrence was determined in a standard manner according to the criteria of Hayward *et al.* (1978) and response to treatment was assessed by UICC criteria (Hayward *et al.*, 1977). Response was classified as either complete/partial (responders) or static/progressive disease (non-responders). Results obtained on a subset of these patients have been described previously (Barnes and Millis, 1995). Patients' details are given in Table I.

### *Histological assessment*

Tumours were typed according to WHO criteria. Histological grade was determined by one of the authors (RRM) according to the method of Bloom and Richardson with modifications as suggested by Elston (1984). (Table I).

**Table I** Patient characteristics

| | |
|---|---|
| Age at diagnosis | |
| Mean | 56 years |
| Range | 27–77 years |
| Tumour size | |
| Mean | 4.01 cm |
| Range | 0–17 cm |
| Menstrual status | |
| Pre | 40 (24%) |
| Peri | 35 (21%) |
| Post | 92 (54%) |
| Other | 3 (1%) |
| Stage | |
| Operable node negative | 41 (24%) |
| Operable node positive | 86 (51%) |
| Nodes unknown | 6 (3%) |
| Locally advanced | 23 (14%) |
| Metastatic | 14 (8%) |
| Histology | |
| Infiltrating ductal | |
| GI | 2 (1%) |
| GII | 92 (54%) |
| GIII | 52 (31%) |
| Infiltrating lobular | 18 (11%) |
| Other | 6 (3%) |
| Nodal status of operable cases | |
| Negative | 41 (32%) |
| 1-3 positive | 40 (32%) |
| >3 positive | 46 (36%) |
| ER status | |
| Cytosol assays | |
| Negative | 44 (26%) |
| Positive | 126 (74%) |
| Histo score | |
| 0–100 | 58 (34%) |
| >100 | 112 (66%) |
| Other methods | |
| Negative | 32 (19%) |
| Positive | 138 (81%) |

### *Oestrogen and progesterone receptor status*

Oestrogen receptor status was determined in two ways. The dextran-coated charcoal LBA was performed at the time of presentation on breast tumour cytosols (King *et al.*, 1979) prepared from either primary tumour tissue ($n=156$) or metastatic lesions ($n=14$). Tissue adjacent to that taken for the cytosol assay was always assessed histologically to ensure that it was representative of the tumour as a whole. Retrospective immunohistochemical assay was performed on routine formalin-fixed paraffin-embedded tissue sections which had been taken from the above. As an attempt was always made to cut sections from a block which contained some normal ducts, inevitably the two assays were frequently carried out on different pieces of tissue. The receptor status in infiltrating carcinomas is generally consistent throughout the tumour and within metastases therefore the fact that different blocks were used in some cases should not be of relevance. PR was only determined retrospectively by immunohistochemistry.

### *Immunohistochemical assay*

Sections of tumour were placed onto APES or vectabond-coated slides and dried for 60 min at 56°C. After blocking endogenous peroxidase activity, sections were placed in a plastic rack in a microwaveable dish containing 0.01 M citrate buffer, pH 6.0. Sections were microwaved for $3 \times 10$ min on 70% power in a 800 watt microwave oven and then removed and allowed to cool to room temperature. After rinsing in distilled water, followed by 0.01 M phosphate-buffered saline pH 7.6 (PBS), sections were covered with 20% normal rabbit serum in PBS to block non-specific binding. ER ID5 monoclonal antibody (Dako) diluted 1:100 in PBS was applied for 1 h at room temperature. After thorough rinsing in PBS ($\times 3$) sections were covered with FAB 2 biotinylated rabbit anti-mouse Ig antibody (Dako) diluted 1:200 with 3% normal human serum in PBS for 30 min. Sections were then rinsed and peroxidase-conjugated streptavidin (Dako) diluted 1:500 in PBS was applied for 30 min. After further rinsing in PBS, peroxidase activity was demonstrated with a solution of hydrogen peroxide/diaminobenzidine and sections were then lightly counterstained with haematoxylin, dehydrated, cleared and mounted.

### *Controls*

Positive and negative controls were always included in every run. A specifically prepared tissue block was used as positive control. This was made from slices of tissue from two tumours with different levels of receptor activity. A negative control, in which primary antibody was omitted, was included for each sample. When selecting tumour material for assay an attempt was always made to use a block which included some normal breast tissue. This acts an excellent internal control as usually at least a few individual normal cells are ER-positive and their presence in a non-staining tumour can confirm genuine ER negativity.

### *Immunohistochemical assay for PR*

Immunohistochemical staining for PR was carried out using essentially the same method as for ER except that the anti-PR antibody NCL-PGR (Novacastra Laboratories Ltd) was used at a dilution of 1 in 40.

### *Assessment of receptor status*

*Ligand-binding assay* A cut-off point of 20 fmol $mg^{-1}$ of cytosol protein was used for the cytosol assay. This was the standard cut-off point in the Unit since, over a long period of time and study, this was found to be the most clinically relevant figure.

*Immunohistochemical assay* Staining was assessed individually by two of the authors (DMB and WH). Disagreement was uncommon (<5% of samples) but when it occurred slides were re-evaluated jointly at a double-headed microscope and agreement reached.

Positive staining was scored in several different ways:

1 Any positive staining at any intensity.
2 Histo score (Kinsel *et al.*, 1989). The intensity of staining of different areas of the section was assessed and allocated a value of 0 (nil), 1 (weak), 2 (distinct) or 3 (strong). The percentage of positive cells in each of the staining categories was also evaluated. The percentage of positive cells was then multiplied by the intensity value and the score of the products added together to give the H score, which thus has a maximum of 300. A cut-off point of 100 was taken to differentiate between positive and negative as was suggested by the authors of the paper first describing the method.
3 The consistency or otherwise of staining was recorded as negative, heterogenous positive or homogeneous positive throughout a tumour.
4 The total proportion of cells staining positively at any intensity was scored as 0 (no cells staining), 1 (when 1–25% cells stained), 2 (when 26–50% cells stained), 3 (when 50–75% cells stained) or 4 (when >75% cells stained).
5 The intensity was scored according to the overall appearance as judged at different powers of magnification, i.e. 0, none (no staining); 1, weak (only visible at high power magnification); 2, moderate (visible at low power magnification); 3, strong (striking even at low power magnification). This was later termed the 'category' score.
6 For some evaluations this system was simplified and the cases subdivided into 'negative' or 'positive'; 'negative' tumours included those with either negative or only weak staining while 'positive' tumours showed moderate or strong staining.

The proportion and intensity scores (methods 4 and 5) were also combined in different ways.

7 The scores were multiplied together to give a range of 1–12 similar to the immunoreactive score (IRS) first described by Remmele and Stegner (1987).
8 These scores were added together to give the 'quick score' as used by Reiner *et al.* (1990), with a range of 2–7.

Staining for PR was only evaluated using the category and simplified category scores.

### *Statistics*

At the outset of the statistical analysis log-rank curves according to the method of Kaplan and Meier (1958) were run for each of the subjective methods of scoring (apart from the histo score) using different combinations of values. The ones chosen for the definitive analysis were those which gave a good separation between patients with different clinical outcomes. The relationships between the different methods of evaluation of ER status and clinical outcome were assessed in various ways. The duration of response to treatment with tamoxifen, i.e. from start of treatment to progression of disease, was determined by log-rank analysis. The chi-squared test was used to examine both the relationship between the results of different methods of ER assay and evaluation as well as the association between receptor status and type of response to treatment. A Cox multivariate analysis was used to find which factors made an independent contribution to the prediction of clinical outcome. The Spearman rank correlation test was used to compare the relationship between type of response and each of the scoring methods for ER using continuous variables.

### Results

Response to tamoxifen, complete or partial was seen in 87/170 (51%) of cases. This high proportion of women showing a positive response is almost certainly due to the prior selection of patients in the study which was based on their known response to hormone treatment. Since the purpose of the study was to relate ER status to response women with unevaluable disease were excluded. The actual percentage of patients with ER-positive tumours depends upon both the method of assay and the method of assessment of staining. The cytosol assay with a cut-off of 20 fmol $mg^{-1}$ found 126/170 (74%) of tumours to be positive. The immunohistochemical assay using the histo score with a cut-off of 100 found 112/170 (65%) of cases to be positive. With all of the other methods of evaluation, 32/170 (19%) of cases were totally negative and 81% showed some level of staining, but the overall number of tumours designated as positive or negative depended on the method of evaluation used. As seen in Table II the proportion of positive cases ranged from 31% (IRS) to 69% (simplified category score) (strong+moderate). Using the simplified category score, 58 cases (34%) were PR-positive. This is a lower proportion than is usually found in an unselected group of patients where approximately 50% of the cases are PR-positive. Examples of positive staining patterns are shown in Figure 1.

The Kaplan–Meier plots in Figure 2 show that all the methods of assay and evaluation of ER gave significant results when used to predict the duration of response. The cytosol assay gave the lowest level of significance ($\chi^2$ 11.91, $P<0.001$). All of the immunohistochemical methods gave chi-squared values of over 30 with the histo score giving the lowest value ($\chi^2$ 30.31 $P<0.0001$) and the category score the highest ($\chi^2$ 49.44 $P<0.0001$).

Similarly when the relationship between different ways of measuring and assessing ER status and type of response was examined, using the chi-squared test the cytosol assay again gave the lowest level of significance with a response rate of 58% in the women with ER-positive tumours ($\chi^2$ 7.89, $P=0.005$) (Table III). The immunohistochemical assay gave results which were highly significant in relation to response irrespective of the method of assessment with chi-squared values ranging from 21.07 for the histo score (64% of ER-positive cases responding) to 31.51 for the category score (69% responders). The addition of PR measured by immunohistochemistry refines the information provided by ER. For example, when tumours were divided into positive and negative using the simplified

**Table II** Relationship between different methods of assessing ER and percentage of ER-positive tumours

| *Assay* | *Cut off (range)* | *ER-positive (%)* |
|---|---|---|
| Any positive staining | ⩾1% (0–100%) | 81 |
| Histo score | >100 (0–300) | 66 |
| Consistency | 100% (<100% or 100%) | 45 |
| Proportion | >75% (0–100%) | 48 |
| Category score (intensity) | ⩾1 (0–3) | 81 |
| Modified category score | ⩾2 (⩽1 or ⩾2) | 69 |
| IRS | 12 (0–12) | 31 |
| Quick score | >5 (0–7) | 48 |
| LBA | >20 fmol $mg^{-1}$ (0–?) | 74 |

**Figure 1** (**a**) Infiltrating lobular carcinoma showing strong positive homogeneous staining for ER in >75% of cells. (**b**) Infiltrating ductal carcinoma showing moderate heterogeneous staining for ER. (**c**) Strong positive staining for ER in a normal breast duct in an ER-negative infiltrating ductal carcinoma. (**d**) Infiltrating ductal carcinoma showing strong heterogeneous staining for PR in an ER-negative tamoxifen-responsive tumour.

category score for both ER and PR, 33/46 (72%) of double positive cases responded. However, 43/71 (61%) of ER-positive PR-negative cases also responded so lack of PR does not preclude a favourable response (Table IV).

*Correlation with other prognostic factors*

The relative influence on time to progression of recognised clinical and histopathological prognostic factors was examined in the multivariate Cox model. Firstly, the model was run on the 128 patients for whom node status was known to assess the relative importance of clinical features excluding ER. The factors included were menstrual status, histological type and grade, tumour size and nodal status; only the first two were significant in both univariate and multivariate models (Table Va). A second model was run on all the patients in the study with the results of the different methods of ER evaluation being treated as continuous variables; also included were menstrual status and histological type and grade. In univariate analysis each factor was found to be significantly related to time to progression. The immunohistochemical methods all gave very similar, highly significant values. Because of the inter-relationship between the different immunohistochemical scoring methods their place in the multivariate model was interchangeable. In this analysis the quick score gave a result which was marginally more significant than the rest. Other features which in the final analysis made independently significant contributions were the cytosol ER assay and histology. (Table Vb). The inclusion of the cytosol assay may at first sight appear surprising but it is well recognised that the greater number of receptor sites the greater is the likelihood of a favourable outcome. Cytosol assays do have the advantage that they measure the total number of sites while the immunohistochemical assay is less sensitive at high levels.

The Spearman rank correlation test showed close inter-relationships between type of response and all of the ER methods of the assay and evaluation. A correlation coefficient of 0.338 was found for the cytosol assay and values ranging from 0.403 for consistency of staining to 0.431 for the quick score were found for the immunohistochemical assays.

*Comparison of results obtained by different methods of evaluation*

There were highly significant associations between the cytosol assay and the immunohistochemical assays, e.g. the cytosol assay compared with the histo score ($\chi^2$ 32.72, $P<0.0001$), the cytosol assay compared with the category score ($\chi^2$ 40.25, $P<0.0001$). There was agreement between the cytosol assay and the immunohistochemical assay, irrespective of the method of evaluation in 137 of the 170 cases (81%). There were 33 cases with discordant results. Thirteen were definitely positive by immunohistochemistry but were negative by the cytosol assay. Eight showed no immunostaining at all but had low positive cytosol values (range 22–54, mean 42 fmol $mg^{-1}$); four of these eight cases had appreciable amounts of positive staining in normal ducts in the sections studied. The remaining 12 cases all showed focal weak (11 cases) or moderate (one case) immunostaining with histo scores less than 100 and had low positive (ten cases) or negative (two cases) cytosol results.

*Comparison of ER status and response*

There were 14 patients with inappropriate ER results who responded to tamoxifen. In one case the immunohistochemistry was negative and the cytosol value was 54 fmol $mg^{-1}$. In

**Figure 2** (**a**) Time to progression as a function of ER status measured by cytosol ligand-binding assay using a cut-off of 20 fmol mg$^{-1}$ cytosol protein. (**b**) Time to progression as a function of ER status determined immunohistochemically and evaluated using the Histo score with a cut-off of 100. (**c**) Time to progression as a function of ER status according to the quick score. (**d**) Time to progression as a function of ER status according to the category score (-ve, negative; w, weak; m, moderate; s, strong).

the other 13 cases cytosol values were negative, three were also ER-negative by immunohistochemistry (but two were PR-positive), the others all showed immunostaining with two being weak, five moderate and three strong stainers.

There were 41 cases with positive ER results both by cytosol assay and by immunohistochemistry (22 with strong staining and 19 with moderate staining), who failed to respond to tamoxifen. All of these patients had one or more of the following poor prognostic features: PR negativity, large tumour size or heavy nodal involvement, the latter two showing that when the tumour burden is overwhelming ER status has little influence on disease outcome.

### Discussion

These results are in agreement with the findings of Goulding *et al.* (1995) and confirm that highly significant clinical information can be obtained from the determination of ER using an immunohistochemical evaluation. Whatever scoring method is used, in this study we show that immunohistochemistry gives superior results to the cytosol assay as it is more closely related to patient outcome. The ligand binding assay was done up to 17 years ago and there has been a general improvement in cytosol assays since the early days. Although it is perhaps unfair to compare methodology of 17 years ago with a current immunohistochemical technique it is also true to say that one of the consequences of the general improvement in cytosol assays has been an increase in the number of cases found to be positive which has resulted in some reduction in the sensitivity of the technique. In the present study we have shown that immunohistochemistry can give results which are clinically relevant, a very important factor now that small tumours are being diagnosed with increasing frequency and no tissue is available for the cytosol assay. Another advantage of the histological method on paraffin-embedded tissue is that the cellularity of the tumour can be taken into consideration when evaluating the staining (Underwood, 1983).

All the methods of scoring used in this study with ER ID5 gave significant results, therefore the method of choice will depend upon further studies into which is the most acceptable and gives the most consistent intra- and inter-observer reproducibility. It is important to select a method which is easy and quick to apply and is reproducible. In our own laboratory, we have had considerable discussions as to whether the proportion or intensity of staining of cells is the best parameter. Immunohistochemists have an inherent scepticism about the significance of intensity of staining as this is known to be affected by staining methods. However strict attention to methods and comparison with controls can overcome this to some extent. Evaluation of the proportion of stained cells on the other hand can also be subjective as well as being affected by technique. Only experience and interlaboratory studies will determine which method is the most appropriate. This study has shown that there is little to choose between the different methods of scoring. The chi-squared test relating ER status to type of response found results with the category score to be the most significant. In the Cox multivariate analysis for time to progression with ER staining treated as a continuous variable the quick score was more significant than the rest.

For over 15 years a quality assurance scheme for ER cytosol assays has been organised within Europe by the European Organisation for the Research and Treatment of Cancer (EORTC). Pilot studies are under way to develop a

**Table III** Relationship between different methods of assay and evaluation and response to tamoxifen

| | *Response* | | | | | |
|---|---|---|---|---|---|---|
| | *Complete/partial* | | *Static/ progressive disease* | *Total* | | |
| | n | % | n | n | % | |
| Cytosol assay | | | | | | |
| 0–20 fmol mg$^{-1}$ | 14 | 32 | 30 | 44 | | $\chi^2=7.89$ |
| >20 fmol mg$^{-1}$ | 73 | 58 | 53 | 126 | 74 | $P=0.005$ |
| Histo score | | | | | | |
| 0–100 | 15 | 26 | 43 | 59 | | $\chi^2=21.07$ |
| >100 | 72 | 64 | 40 | 112 | 66 | $P<0.0001$ |
| Consistency of staining | | | | | | |
| Negative | 4 | 13 | 28 | 32 | | $\chi^2=26.88$ |
| Heterogeneous | 32 | 52 | 30 | 62 | | $P<0.0001$ |
| Homogeneous | 51 | 67 | 28 | 76 | 45 | |
| Proportion | | | | | | |
| Negative | 4 | 13 | 28 | 32 | | $\chi^2=28.32$ |
| ⩽75% | 28 | 48 | 41 | 57 | | $P<0.0001$ |
| >75% | 55 | 68 | 14 | 81 | 48 | |
| IRS | | | | | | |
| Negative | 4 | 13 | 28 | 32 | | $\chi^2=29.19$ |
| 1–11 | 45 | 52 | 41 | 86 | | $P<0.0001$ |
| 12 | 38 | 73 | 14 | 52 | 31 | |
| Quick score | 4 | 13 | 28 | 32 | | $\chi^2=27.48$ |
| Negative | 28 | 50 | 28 | 56 | | $P<0.0001$ |
| 1–5 | 55 | 67 | 27 | 82 | 48 | |
| 6–7 | | | | | | |
| Category score | 4 | 13 | 28 | 32 | | $\chi^2=31.51$ |
| Negative | 7 | 34 | 14 | 21 | | $P<0.0001$ |
| Weak | 33 | 61 | 22 | 55 | | |
| Moderate | 43 | 69 | 19 | 62 | 36 | |
| Strong | | | | | | |
| Simplified category score | | | | | | |
| Negative and weak | 11 | 21 | 42 | 53 | | $\chi^2=26.79$ |
| Moderate and strong | 76 | 65 | 41 | 117 | 69 | $P<0.0001$ |

**Table IV** Relationship between combined ER and PR status (simplified category score; negative and weak *vs* moderate and strong) and response to tamoxifen treatment

| | *Response* | | | | | |
|---|---|---|---|---|---|---|
| | *Complete/ partial* | | *Static/ progressive disease* | *Total* | | |
| | n | % | n | n | % | |
| ER/PR status | | | | | | |
| ER– PR– | 8 | 20 | 33 | 41 | 24 | $\chi^2=30.03$ |
| ER– PR+ | 3 | 20 | 9 | 12 | 7 | $P<0.0001$ |
| ER+ PR– | 43 | 61 | 28 | 71 | 42 | |
| ER+ PR+ | 33 | 72 | 13 | 46 | 27 | |
| | 87 | 51 | 83 | 170 | | |

**Table V** Cox multivariate analyses showing which factors are significantly related to the prediction of clinical outcome

| | *Univariate* | | *Multivariate* | |
|---|---|---|---|---|
| | $\chi^2$ | P-*value* | $\chi^2$ | P-*value* |
| a | | | | |
| Menstrual status | 6.6 | 0.01 | 4.9 | 0.03 |
| Histology | 7.7 | 0.006 | 7.7 | 0.006 |
| Tumour size | 3.5 | 0.06 | | |
| Nodal status | 0.3 | 0.6 | | |
| b | | | | |
| Menstrual status | 10.8 | 0.001 | 3.7 | 0.06 |
| Histology | 8.0 | 0.005 | 4.7 | 0.03 |
| ER cytosol | 23.8 | <0.001 | 5.9 | 0.02 |
| IRS | 37.3 | <0.001 | | |
| Quick score | 41.8 | <0.001 | 41.8 | <0.0001 |
| H score | 36.7 | <0.001 | | |
| Simplified category score | 38.7 | <0.001 | | |
| Category score | 39.9 | <0.001 | | |

similar scheme for ER on formalin-fixed paraffin-embedded tissue. In the UK the National External Quality Assessment Scheme (NEQAS) for quality control in diagnostic immunohistochemistry is well established and this scheme has recently included ER in their assessments and it is of interest to note that improvements in the quality and consistency of staining were already apparent in the second round results.

The correct cut-off point for ER assays has been debated over the years. In resolving this issue the reason for doing the assay must always be borne in mind. Is ER being measured to identify women who are likely to benefit from endocrine therapy as either an adjuvant therapy for early disease or as treatment for metastatic disease or is it being used as a marker of prognosis? When considering prediction of response in the case of metastatic disease, it should be remembered that only 30 to 40% of all patients will respond to hormone treatment. This is a much lower level than the incidence of ER positivity. In the early days of the assay this discrepancy was not so marked but as more was learnt about the extreme care which needs to be taken over storage conditions and assay procedures, the sensitivity of the assays has improved and the proportion of patients found to have ER-positive tumours has increased. But does this make the results more clinically relevant?

It should also be noted that both the absolute amount of ER and the proportion of ER-positive tumours increases with age. In order to take account of this Jensen and DeSombre (1993) have suggested that tumours should be described as ER-rich and ER-poor. Maybe we should revert to this for immunohistochemical results. Our results show that the more positive staining there is the greater the likelihood of a favourable response and with less staining fewer patients will respond.

When it became obvious that not all ER-positive tumours respond to hormone treatment other indicators of response were sought. Horwitz and McGuire (1975) suggested that PR, an oestrogen-induced protein, should also be measured. The theory was that the coexpression of PR would indicate that the ER in the tumour was functionally active. In practice, the measurement of the two receptors does improve predictive accuracy: ER-positive PR-positive tumours have approximately 80% likelihood of responding whereas double-negative tumours have a less than 10% likelihood of response (Stewart *et al.*, 1982). However, half of the ER-positive PR-negative tumours also show a favourable response. Therefore if, as has been suggested, only PR status was evaluated a significant number of patients who would respond might be denied endocrine treatment. Our results in this study are consistent with these findings. More recently other oestrogen-induced proteins such as cathepsin D and PS2 have been studied but neither of these alone or in combination with ER have proved to be outstandingly successful in refining response prediction.

The use of immunohistochemistry for the measurement of ER has many advantages, some of which have been outlined above. Not least is the great reduction in cost of the assays which have followed the introduction of the newer antibodies. In the current times of financial stringency, this factor is of increasing relevance. The assay is now easier to do and the results should become more reliable with improvements in quality control. It is likely that ER will have a continuing role in the management of patients with breast cancer and interlaboratory consistencies will improve as agreement is reached as to the best method of scoring. Nevertheless, the assays should only be carried out when the results are going to be of either practical clinical or research value.

### References

AL SAATI T, CLAMENS S, COHEN-KNAFO E, FAYE JC, PRATS H, COINDRE JM, WAFFLART J, CAVERIVIERE, BAYARD F AND DELSOL G. (1993). Production of monoclonal antibodies to human oestrogen receptor protein (ER) using recombinant ER (RER). *Int. J. Cancer*, **55**, 651–654.

ANDERSEN J, THORPE SM, KING WJ, ROSE C, CHRISTENSEN I, RASMUSSEN BB AND POULSEN HS. (1990). The prognostic value of immunohistochemical estrogen receptor analysis in paraffin-embedded and frozen sections versus that of steroid-binding assays. *Eur. J. Cancer*, **26**, 442–449.

BARNES DM AND MILLIS RR. (1995). Oestrogen receptors: the history, the relevance and the methods of evaluation. In *Progress in Pathology* Vol 2. Kirkham N, Lemoine N (eds). Churchill Livingtsone: London.

BOBROW LG, HAPPERFIELD LC, GREGORY WM, SPRINGALL RD AND MILLIS RR. (1994). The classification of ductal carcinoma *in situ* and its association with biological markers. *Semin. Diagn. Pathol.*, **11**, 199–207.

ELSTON CW. (1984). The assessment of histological differentiation in breast cancer. *Aust. NZ. J. Surg.*, **54**, 11–15.

GREENE GL, NOLAN C, ENGLER JP AND JENSEN EV. (1980). Monoclonal antibodies to human estrogen receptor. *Proc. Natl Acad. Sci. USA*, **77**, 5115–5119.

GOULDING H, PINDER S, CANNON P, PEARSON D, NICHOLSON R, SNEAD D, BELL J, ELSTON CWE, ROBERTSON JF, BLAMEY RW AND ELLIS IO. (1995). A new immunohistochemical antibody for the assessment of estrogen receptor status on routine formalin-fixed tissue samples. *Hum. Pathol.*, **26**, 291–294.

HAYWARD JL, CARBONE PP, HEUSON JC, KUMAOKA S, SEGALOFF A AND RUBENS RD. (1977). Assessment of response to therapy in advanced breast cancer. *Cancer*, **39**, 1289–1294.

HAYWARD JL, MEAKIN JW AND STEWARD HJ. (1978). Assessment of response and breast cancer. *Semin. Oncol.*, **5**, 445–449.

HORWITZ KB, MCGUIRE WL, PEARSON OH AND SEGALOFF A. (1975). Predicting response to endocrine therapy in human breast cancer: a hypothesis. *Science*, **189**, 726–727.

JENSEN EV AND DESOMBRE ER. (1993). Steroid hormone binding and hormone receptors In *Cancer Medicine*. 3rd edn. Holland JF, Frie E III, Bast RC Jr, Kufe DW, Morton DL, Weichselbaum RR (eds). pp. 815–823. Lea and Febinger: Philadelphia.

KAPLAN EL AND MEIER P. (1958). Non-parametric estimation from incomplete observations. *J. Am. Stat. Assoc.* **53**, 457–481.

KING RJB, REDGRAVES, HAYWARD JL, MILLIS RR AND RUBENS RD. (1979). The measurement of receptors for oestradiol and progesterone in human breast tumors. In *Steroid Receptor Asssays in Breast Tumors: Methodological and Clinical Aspects.* King RJB (ed) pp. 55–73. Alpha-Omega: Cardiff.

KING WL, DESOMBRE ER, JENSEN EV AND GREENE GL. (1985). Comparison of immunocytochemical and steroid-binding assays for estrogen receptor in human breast tumors. *Cancer Res.*, **45**, 293–304.

KINSEL LB, SZABO E, GREENE GL, KONRATH J, LEIGHT GS AND MCCARTY KS JR. (1989). Immunocytochemical analysis of estrogen receptors as a predictor of prognosis in breast cancer patients: comparison with quantitative biochemical methods. *Cancer Res.*, **49**, 1052–1056.

KORENMAN SG AND DUKES BA. (1970). Oestrogen receptors in human breast cancer: the Edinburgh experience. *J. Clin. Endocrinol.*, **30**, 659–664.

PRESS MF AND GREENE GL. (1988). Localization of progesterone receptor with monoclonal antibodies to the human progestin receptor. *Endocrinology*, **122**, 1165–1175.

REINER A, NEUMEISTER B, SPONA J, REINER G, SCHEMPER M AND JAKESZ R. (1990). Immunocytochemical localization of estrogen and progesterone receptor and prognosis in human primary breast cancer. *Cancer Res.*, **50**, 7057–7061.

REMMELE W AND STEGNER HE. (1987). Immunhistochemischer nachweis von ostrogenerezeptoren (ER-ICA) im mammakarizinomgewebe: vorschlag zur einheitlichen bewertung des untersuchungsbefundes. *Frauenarzt*, **28**, 41–43.

SACCANI JOTTI G, JOHNSTON SRD, SALTER J, DETRE S AND DOWSETT M. (1994). Comparison of new immunohistochemical assays for oestrogen receptor in paraffin wax embedded breast carcinoma tissue with quantitative enzyme immunoassay. *J. Clin. Pathol.*, **47**, 900–905.

STEWART J, ROGER K, HAYWARD J AND RUBENS R. (1982). Estrogen and progesterone receptors: correlation of response rates, site and timing of receptor analysing. *Br. Cancer Res. Treat.*, **2**, 243–250.

TAYLOR CR, SHI S, CHAIWUN B, YOUNG L, ASHRAF IMAM S AND COTE RJ. (1994). Strategies for improving the immunohistochemical staining of various intranuclear prognostic markers in formalin-paraffin sections. *Hum. Pathol.*, **25**, 263–270.

UNDERWOOD JCE. (1983). Oestrogen receptors in human breast cancer: review of histopathological correlates and critique of histochemical methods. *Diagn. Histopathol.*, **6**, 1–22.

VERONESE SM, BARBARESCHI M, MORELLI L, ALDOVINI D, MAURI FA, CAFFO O, GAMBACORTA M AND PALMA PD. (1995). Predictive value of ER1D5 antibody immunostaining in breast cancer. a paraffin-based retrospective study of 257 cases. *Appl. Immunohistochem.*, **3**, 85–90.